# Association between temporal patterns of baroreflex sensitivity after traumatic brain injury and prognosis: a preliminary study

**DOI:** 10.1007/s10072-022-06579-7

**Published:** 2023-01-07

**Authors:** Agnieszka Uryga, Magdalena Kasprowicz, Małgorzata Burzyńska, Agnieszka Kazimierska, Marek Czosnyka, Nathalie Nasr

**Affiliations:** 1 Department of Biomedical Engineering, Faculty of Fundamental Problems of Technology, Wroclaw University of Science and Technology, Wybrzeze Wyspianskiego 27, 50-370 Wroclaw, Poland; 2 Department of Anaesthesiology and Intensive Care, Wroclaw Medical University, Wroclaw, Poland; 3 Brain Physics Laboratory, Division of Neurosurgery, Department of Clinical Neurosciences, Addenbrooke’s Hospital, University of Cambridge, Cambridge, UK; 4 Institute of Electronic Systems, Faculty of Electronics and Information Technology, Warsaw University of Technology, Warsaw, Poland; 5 Department of Neurology, Poitiers University Hospital, Poitiers, France

**Keywords:** Autonomic nervous system, Traumatic brain injury, Baroreflex sensitivity, Pressure reactivity index, Prognosis

## Abstract

**Introduction:**

Traumatic brain injury (TBI) may lead to an increase in intracranial pressure (ICP) as well as impairment of cerebral vascular reactivity and the autonomic nervous system. This study aimed to investigate individual patterns of changes in baroreflex sensitivity (BRS) along with the assessment of pressure reactivity index (PRx) and ICP after TBI.

**Materials and methods:**

Twenty-nine TBI patients with continuous arterial blood pressure (ABP) and ICP monitoring were included. BRS was calculated using the sequential cross-correlation method. PRx was estimated using slow-wave oscillations of ABP and ICP. Outcome was assessed using the Glasgow Outcome Scale.

**Results:**

Pooled data analysis of the lower breakpoint during the week that followed TBI revealed that BRS reached a minimum about 2 days after TBI. In patients with good outcome, there was a significant increase in BRS during the 7 days following TBI: *r*_*p*_ = 0.21; *p* = 0.008 and the temporal changes in BRS showed either a “U-shaped” pattern or a gradual increase over time. The BRS value after 1.5 days was found to be a significant predictor of mortality (cut-off BRS = 1.8 ms/mm Hg; AUC = 0.83). In patients with poor outcome, ICP and PRx increased while BRS remained low.

**Conclusions:**

We found an association between temporal patterns of BRS and prognosis in the early days following TBI. Further research in a larger cohort of patients is needed to confirm the weight of these preliminary observations for prediction of prognosis in TBI patients.

**Supplementary Information:**

The online version contains supplementary material available at 10.1007/s10072-022-06579-7.

## Introduction

Traumatic brain injury (TBI) may lead to several secondary cerebral changes, including cerebral edema and ischemic complications due to a reduction in cerebral perfusion pressure resulting from impairment of blood flow regulation associated with systemic disorders [1–4]. An important factor responsible for cardiovascular homeostasis is the autonomic nervous system (ANS). Impairment of ANS function may lead to systemic complications, cardiac disorders, and also immune system depression; these adverse effects have been observed in patients with severe and mild TBI [3, 5, 6]. Moreover, impairment of ANS function is associated with poor outcome and increased mortality in brain injury patients [7–9]. The presence of baroreflex impairment and a decrease in heart rate variability (HRV) in acute brain injury has led to the hypothesis that in acute cerebrovascular insult there is an uncoupling between the cardiovascular and autonomic nervous systems [10].

Previous studies have also shown that ANS activity may be altered during an increase in intracranial pressure (ICP) which is a common occurrence in severe TBI patients [11, 12]. An increase in ICP may lead to dysautonomia, characterized by severe increases in heart rate (HR), respiratory rate, temperature, and blood pressure, as well as excessive sweating [13]. However, because of a limited understanding of its pathophysiology, the prognostic significance of dysautonomia in TBI remains unclear [5]. Arterial baroreflex sensitivity (BRS) is one of the metrics that describe the influence of the ANS on the cardiovascular system [14]. Closed-loop models of the interactions between blood pressure and heart rate variations allow for the estimation of BRS which quantifies feedback effects of changes in pressure on heart rate while also considering the feedforward effects of heart rate on blood pressure [15]. However, BRS cannot be viewed solely as the heart rate response to arterial blood pressure changes, as transfer function analysis of baroreflex suggests that low frequency changes in arterial blood pressure result in part from the sympathetic component of baroreflex, hypothesizing a resonant system characterized by self-sustained oscillations of arterial blood pressure [16]. BRS can be easily assessed at the bedside in the intensive care unit (ICU) using continuous monitoring of ABP and HR which are routinely measured in clinical practice. It has been shown that a rise in ICP is related to a significant increase in BRS up to the upper breakpoint of the ICP amplitude–pressure characteristic (i.e., the relationship between pulse amplitude of ICP and mean ICP) where mean ICP is at extreme levels, after which ICP continues to rise while BRS decreases [9]. However, the association between the dynamics of BRS trends measured day to day and TBI prognosis is not known.

The understanding of factors that influence TBI prognosis could be improved by the assessment of individual day-to-day changes in different physiological parameters in addition to reporting averaged values. The study of Papaioannou et al. has shown a progressive daily increase in the transfer function gain between systolic blood pressure and HR in TBI patients who survived [17]. The importance of determining individual time trends of ANS metrics was also raised by Sykora et al. [18]. In their work about ANS disturbance in TBI patients, the authors showed that low average values of BRS (estimated using the cross-correlation method [19]) and an increase in the relative power in the high-frequency band of HRV were predictors of mortality after TBI, independently from ICP or PRx. 

Here, we investigated individual patterns of BRS, estimated continuously in the time domain using the cross-correlation method, within the first 7 days after TBI, taking into account alterations in mean ICP and cerebrovascular reactivity.

## Materials and methods

### Study population

**Fig. 1:**
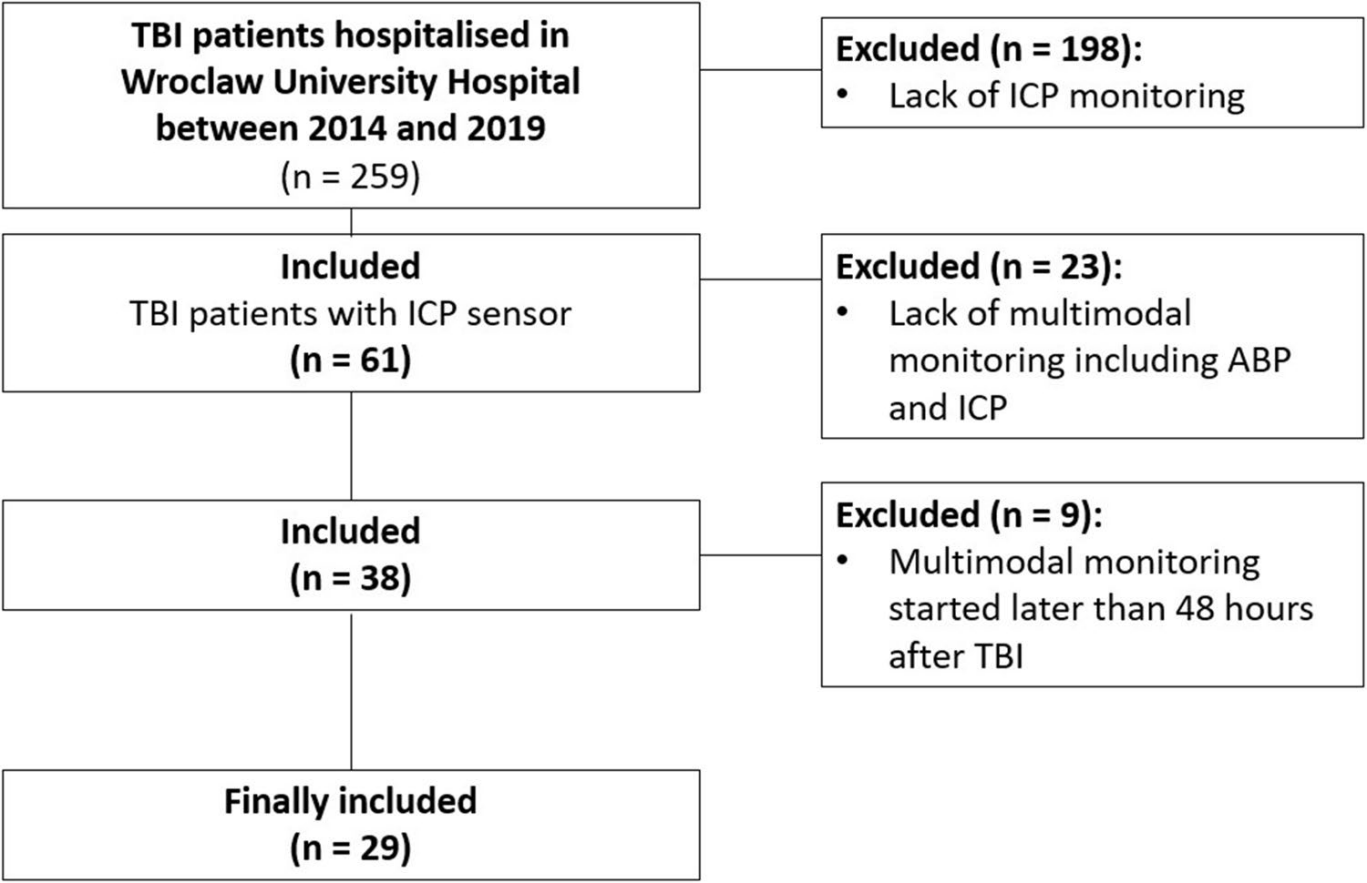
Flow chart of study design. Abbreviations: TBI, traumatic brain injury; ABP, arterial blood pressure; ICP, intracranial pressure

This study was a retrospective single-center analysis of data collected at Wroclaw University Hospital (Wroclaw, Poland) from patients admitted to the ICU for TBI management between January 2014 and December 2019. The study was approved by the local Ethics Committee (approval no. KB-624/2014). Inclusion criteria were as follows: age ≥ 18 years, diagnosis of TBI, and multimodal monitoring including arterial blood pressure (ABP) and ICP. The exclusion criteria were: continuous monitoring not available or available monitoring started later than 48 h after TBI. The flow chart showing the exclusion criteria and the study’s experimental design is presented in Fig. 1. Patients were treated according to guidelines applicable at the time of admission [20]. Patients received analgesic drugs (propofol and fentanyl), anti-edema drugs if ICP was higher than 22 mm Hg (mannitol and furosemide), and circulatory medications (noradrenaline) to maintain cerebral perfusion pressure at 60–70 mm Hg. The partial pressure of carbon dioxide (PaCO_2_) in arterial blood was within the normal range of 35–45 mm Hg in all patients.

### Signal monitoring

ICP was measured invasively using an intraparenchymal sensor (Codman MicroSensor ICP Transducer, Codman & Shurtleff, MA, USA) inserted into the frontal cortex. ABP was measured in the radial or femoral artery using standard monitoring kits (Baxter Healthcare, Cardiovascular Group, Irvine, CA, USA). The signals were recorded with a sampling frequency of 200 Hz using Intensive Care Monitor (ICM+) software (Cambridge Enterprise Ltd, Cambridge, UK) during the patient’s ICU stay. In patients who required craniectomy, the monitoring was stopped after surgery. All artifacts in the recordings were selected either manually or by custom-written algorithms, and further analyses were performed only on the representative parts of the signals.

### Pressure reactivity index

The pressure reactivity index (PRx) was calculated as the Pearson linear correlation coefficient between slow waves in the ABP and ICP signal. First, the signals were averaged over 10-s intervals to isolate the slow changes, and then the correlation coefficient was assessed in 5-min moving average windows updated every 10 s [21]. PRx is expressed in arbitrary units (a.u.). Positive PRx indicates impaired cerebral autoregulation, as it describes passive transmission of fluctuations from ABP to ICP [22].

### Baroreflex sensitivity

Baroreflex sensitivity (BRS) was assessed in the time domain based on the sequential cross-correlation method proposed by Westerhof et al. [19] using built-in functions of ICM+ software. It was calculated as the slope of the regression line between 10-s segments of the systolic peak-to-peak interval and the corresponding systolic pressure time series derived from the ABP signal. Due to the variability of systolic pressure and interbeat interval, resulting in varying delay between the time series, the algorithm considers time shifts of 0–5 s, and the cross-correlation function is used to obtain the maximum correlation coefficient considering the unknown time shift between the series. It has been shown that the variability of xBRS, defined as the coefficient of variation of BRS (SD/mean), is almost constant during recording, irrespective of the mean level of BRS. Moreover, in young, healthy adults the optimal delay *τ* is 0 or 1 s, but it may reach 2 or 3 s depending on the degree of vagal versus sympathetic dominance [23]. In our study, the dominant time delay in the total group was 1 s (48% of patients) or 0 s (28% of patients).

### Analysis of time trends

Data from the day of trauma (or the next day, depending on the day of surgery, but starting no later than 48 h post-injury) through day 7 were used for the calculation of analyzed parameters based on a previous study which showed that in TBI patients the main prognostic parameters (e.g., ICP-derived indices) are different between survivors and non-survivors only during the first 7 days post-injury [24]. For mortality prediction and the assessment of the relationship between BRS and ICP, a non-overlapping 12-h window was used to determine the averaged values of the parameters in each half-day period.

### Outcome

Patients’ follow-up was assessed using the Glasgow Outcome Scale (GOS) scores at discharge from the hospital. Outcome was classified as poor (GOS 1–3) or good (GOS 4–5). Mortality was defined as 30-day all-cause mortality after discharge from the hospital. Patients were categorized into the surviving group (alive after 30 days) and the non-surviving group (dead within 30 days) based on the 30-day outcome.

### Statistics

The normality of data distributions was assessed using the KolmogorovSmirnov test with Lilliefors correction. Non-parametric tests were applied in further analyses due to the rejection of the normality hypothesis for most of the analyzed indices. Differences in median values categorized by any dichotomized criteria defined in this study were tested using the Mann–Whitney *U* test or using Pearson’s chi^2^ test (or Fisher exact test) for non-numeric data. The significance of changes in BRS and PRx between two consecutive 12-h windows (e.g., between values derived from the first 12 h and 12–24 h) was assessed using Wilcoxon signed-rank test. The relationships between the number of days elapsed since TBI (predictors) and BRS values (responses) were calculated using multiple linear regression analysis, with subjects treated as categorical factors using dummy variables (concerning the inter-subject variability), where the partial correlation coefficient (*r*_*p*_) between analyzed variables was estimated as recommended by Bland and Altman [25, 26]. To investigate the average time from TBI to the nadir of BRS (lower BRS breakpoint) and the peak of PRx (upper PRx breakpoint) during the first 7 days post-injury, the individual extremum of BRS and PRx for each patient was determined. Then, we calculated Tukey median estimator in the total group and presented the results as a bag plot. Threshold values of BRS and PRx for mortality prediction were estimated using receiver operating characteristic (ROC) curves and assessed using the area under the curve (AUC). Based on the results of the ROC curve analysis, a model for early prediction of mortality was proposed. BRS and PRx, which had significant AUC at the earliest time window, and GCS at admission <= 8 [27, 28] were chosen as binary classifiers in early prediction of mortality. The “unfavorable” pattern of changes in BRS over time in the first 7 days after TBI and mean PRx > 0.3 (averaged from the 7 days following TBI) [29] were determined as binary classifiers for late prediction of mortality. Accuracy analysis of the proposed binary classification of mortality risk was performed using MedCalc Software Ltd. Spearman’s rank correlation (*r*_*S*_) was used to examine the relationships between BRS and ICP values. All group-averaged data are presented as median ± IQR unless stated otherwise.

## Results

### Patient characteristics

The cohort consisted of 21 men and 8 women aged 33 ± 22 years. All patients included in the analysis were free from comorbidities or concomitant treatment. Patients suffered predominantly from severe TBI (Glasgow Coma Scale [GCS] score of 8 or less [90%]). A minority of patients were classified as having moderate TBI (GCS 9 to 12 [10%]). The patients’ median GCS score was 7 ± 3. The 30-day mortality rate was 28%. Thirteen patients (45%) had a poor treatment outcome. Detailed patient characteristics are presented in Table 1.

**Table 1:** Clinical characteristics of traumatic brain injury (TBI) patients included in the study (29 patients)

Parameter	Value
Age [years]	33 ± 22
Gender: male	21 (72%)
Gender: female	8 (28%)
GCS	7 ± 3
GCS: mild TBI (13–15)	0
GCS: moderate TBI (9–12)	3 (10%)
GCS: severe TBI (8 or less)	26 (90%)
Craniotomy	1 (3%)
EVD or CSF drainage	1 (3%)
Evacuation of epidural and subdural hematomas	5 (17%)
Marshall scale	3 ± 3
Rotterdam scale	4 ± 3
Pupil anisocoria	9 (31%)
Pupils non-reactive	7 (24%)
Subdural hematoma	11 (38%)
Epidural hematoma	4 (14%)
Cerebral hematoma	7 (24%)
Edema/cerebral contusion	16 (55%)
Axonal trauma	4 (14%)
SAH	8 (28%)
Isolated head trauma	9 (31%)
GOS	3 ± 1
GOS: grade 1	8 (28%)
GOS: grade 2	0
GOS: grade 3	5 (17%)
GOS: grade 4	12 (41%)
GOS: grade 5	4 (14%)
30-day mortality	8 (28%)

*EVD* external ventricular drainage, *CSF* cerebrospinal fluid, *SAH* subarachnoid hemorrhage, *GCS *Glasgow Coma Scale, *GOS* Glasgow Outcome Scale

Data are presented as median ± interquartile range or as the number of patients (% of the total group)

### Long time trends in BRS vs. outcome

**Fig. 2:**
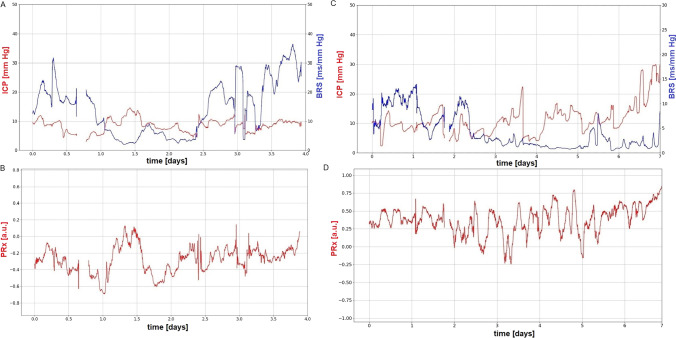
Illustrative examples of temporal patterns in baroreflex sensitivity (BRS; blue line, upper panels), intracranial pressure (ICP; red line, upper panels), and pressure reactivity index (PRx; red line, bottom panels) in **A**–**B**): 20-year-old man who survived with Glasgow Outcome Scale score of 4; **C**–**D** 69-year-old woman who died. Patients who survived presented a gradual increase in BRS after day 2. In contrast, patients who died demonstrated a gradual decrease in BRS after day 2.5

**Fig. 3:**
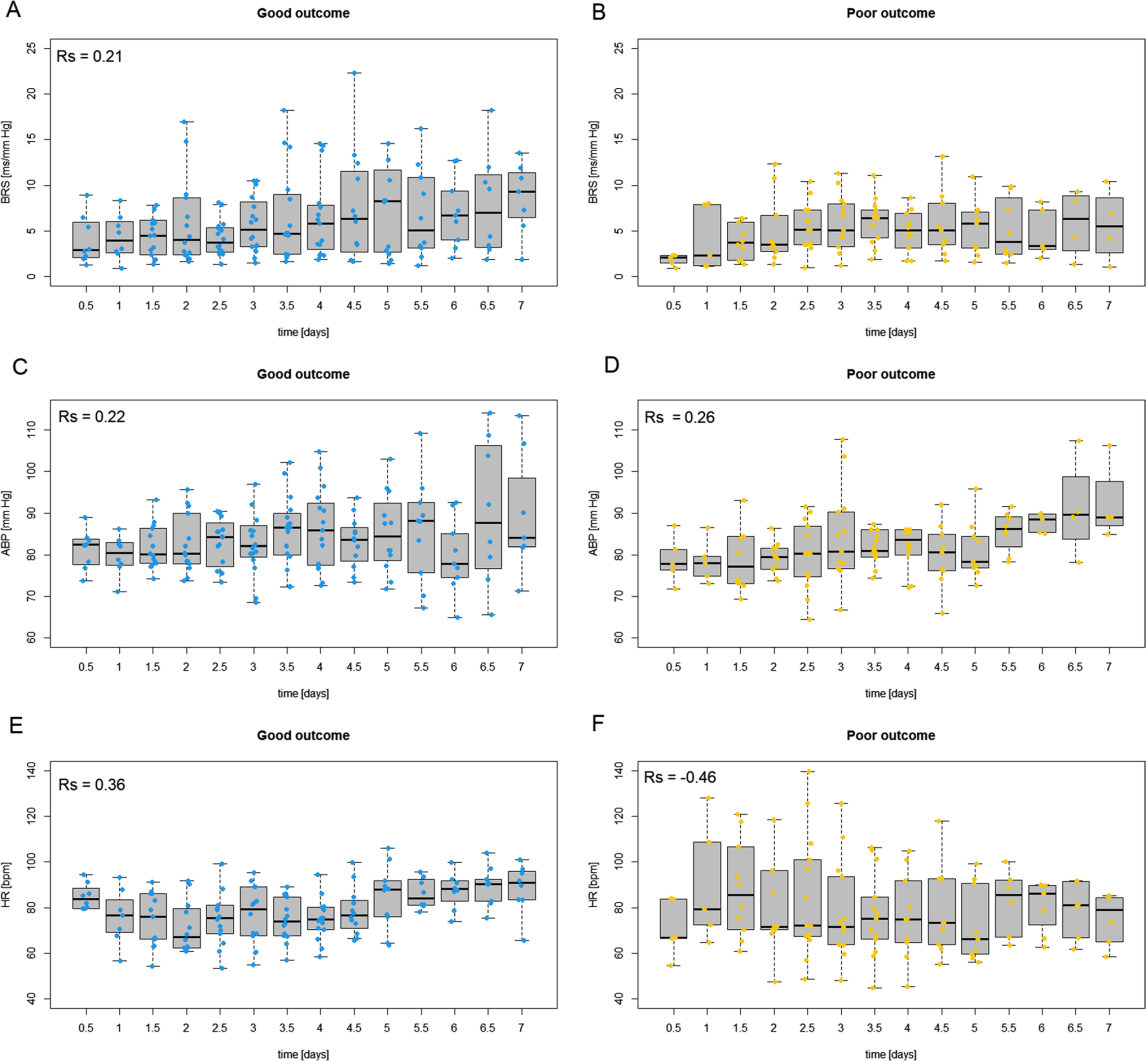
Median values of baroreflex sensitivity (BRS), arterial blood pressure (ABP), and heart rate (HR) calculated in consecutive non-overlapping 12-h windows during the first 7 days following traumatic brain injury in the group with good outcome (blue dots; sub-panels: **A**, **C**, **E**) and poor outcome (orange-yellow dots; sub-panels: **B**, **D**, **F**). Group-averaged data are presented as medians (central thick black lines), interquartile ranges (gray boxes), and min-max values (whiskers). Individual patients are presented as dots

In the group of patients with good outcome, BRS in the first 7 days after TBI presented a “U-shaped” curve or gradually increased over time. An example of the temporal pattern of BRS in a patient with good outcome is presented in Fig. 2A. The partial correlation analysis showed a significant but weak increase in BRS in the first 7 days after TBI in patients with good outcome *r*_*p*_ = 0.21, *p* = 0.008; see Fig. 3A. This was not observed in patients with poor outcome (see Fig. 3B).

### Long time trends in ABP and HR vs. outcome

The time trends in ABP and HR in the first 7 days in the group with poor and good outcomes are presented in Fig. 3C–F. The partial correlation analysis showed an increase in ABP in patients with good outcome *r*_*p*_ = 0.22, *p* = 0.008 (Fig. 3C) and with poor outcome *r*_*p*_ = 0.26, *p* = 0.006 (Fig. 3D). Moreover, in patients with good outcome HR increased *r*_*p*_ = 0.36, *p* < 0.001 (Fig. 3E), whereas in patients with poor outcome HR decreased *r*_*p*_ = −0.46, *p* < 0.001 (Fig. 3F).

### Temporal patterns in BRS vs. mortality

In patients who died, we observed three possible scenarios identified as “unfavorable” patterns: BRS in the days following injury reached an upper breakpoint and then dropped; BRS gradually decreased over time; or BRS remained low (< 5 ms/mm Hg). An example of the BRS time trend in a patient who died is presented in Fig. 2C.

### Temporal patterns in ICP and PRx

We observed that in patients with good outcome, in addition to an increase in BRS over time, ICP and PRx were in the normal range (ICP < 22 mm Hg [20] and PRx < 0 [29]; Fig. 2A, B). Conversely, in patients with poor outcome, ICP and PRx increased while BRS remained low, indicating a lack of significant response to ICP stimulation (Fig. 2C, D).

### The lower breakpoints of BRS and PRx

**Fig. 4:**
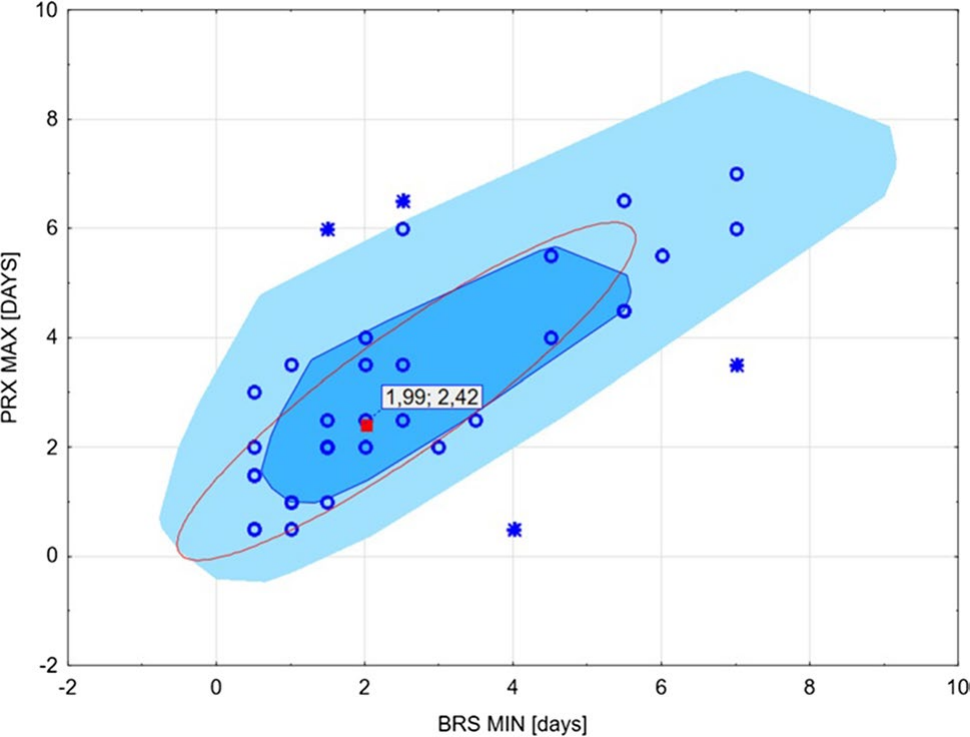
The “breakpoint” analysis using Tukey’s bag plot: graphical representation of the day when baroreflex sensitivity (BRS) reached minimum value (BRS MIN), and pressure reactivity index (PRx) was the highest (PRx MAX) in the full group of traumatic brain injury (TBI) patients. BRS reached the minimum value on day 2 after TBI, while PRx was the highest on day 2.5 after TBI. The following notations are used in the Tukey plot: the red square is the Tukey median which is the point with maximum depth; the bag (dark blue) is the depth region (which is a convex polygon) that contains 50% of the points with the largest depth; the fence (light blue) was calculated as depth regions; points that are outside the outer fence are marked as outliers with asterisks

Tukey’s bag plot analysis of the days when BRS was at its lowest and PRx was at its highest (lower/upper breakpoints of the parameters) showed that on average, BRS reached a minimum value about 2 days after TBI, while PRx was at its highest on day 2.5 after TBI (Fig. 4). The values of BRS, ICP, and PRx in the first days after TBI are presented in Supplementary Table 1.

### The association of daily changes in BRS and PRx with mortality

During the first 1.5 days after injury, the BRS value was significantly lower (3.58 ± 3.75 ms/mmHg) than in days 2–7 (5.40 ± 4.53 ms/mmHg; *p* = 0.024). The value of BRS at 1.5 days was found to be significantly associated with mortality (cut-off BRS = 1.8 ms/mmHg; AUC = 0.83; *p* < 0.001). PRx in days 1–3 was found to be significantly associated with mortality, with threshold values ranging from 0.17 to 0.35 (see Supplementary Table 2).

### Early prediction of mortality

Based on the results of ROC curve analysis, we proposed a model for early prediction of mortality. The patient was classified as “at risk” of death if BRS was <= 1.8 ms/mm Hg or PRx was >= 0.17 when estimated at 1.5 days after TBI. Both models achieved moderate accuracy; however, the BRS-based classifier had better specificity (see Table 2). The model using GCS <= 8 had high sensitivity (all death cases were marked as “at risk”), but poor specificity (see Table 2).

**Table 2:** Performance of the models for early prediction of mortality based on Glasgow Coma Scale (GCS) value <= 8 (severe traumatic brain injury) at admission, baroreflex sensitivity (BRS) <= 1.8 ms/mm Hg or pressure reactivity index (PRx) >= 0.17 at 1.5 days after injury and the models for late prediction of mortality based on ‘unfavorable pattern’ of BRS or PRx > 0.3 estimated from total seven days after injury

Early prediction of mortality				Late prediction of mortality	
	GCS <= 8	BRS <= 1.8	PRx >= 0.17	“Unfavorable pattern” of BRS	PRx > 0.3
SE performance metric	100.00 (63.06–100.00)	60.00 (14.66–94.73)	60.00 (14.66–94.73)	87.50 (47.35–99.68)	62.50 (24.49–91.48)
SPE	14.29 (3.05–36.64)	84.21 (60.42–96.62)	72.22 (46.52–90.31)	80.95 (58.09–94.55)	95.24 (76.18–99.88)
PPV	30.77 (27.18–34.64)	50.00 (22.08–77.92)	37.50 (17.60–62.77)	63.64 (41.09–81.45)	83.33 (40.68–97.33)
NPV	100.00	88.98 (72.88–95.97)	86.67 (68.15–95.18)	94.44 (72.87–99.08)	86.96 (73.05–94.25)
ACC	37.93 (20.69–57.74)	79.17 (57.85–92.87)	69.57 (47.08–86.79)	82.76 (64.23–94.15)	86.21 (68.34–96.11)

*SE* sensitivity, *SPE* specificity, *PPV* positive predictive value, *NPV* negative predictive value, *ACC* accuracy

Data are presented as mean (95% confidence interval)

### Late prediction of mortality

The model for late prediction of mortality was based on an “unfavorable pattern” of BRS or PRx > 0.3 estimated from the whole 7 days after injury. The performance of those two predictors is presented in Table 2. Both metrics were found to be good and comparable late discriminators of mortality.

### Relationship between BRS and ICP

In our study, we found a significant correlation between ICP and BRS when estimated using data from the first 7 days (*r*_*S*_ = 0.49; *p* = 0.007), i.e., an increase in ICP was associated with an increase in BRS. This observation was also found in the following days after TBI: day 1, *r*_*S*_ = 0.52; *p* = 0.004; day 3.5, *r*_*S*_ = 0.66; *p* < 0.001; and day 4:*r*_*S*_ = 0.42; *p* = 0.039.

## Discussion

The observation that baroreflex function is dynamic and may change spontaneously under both physiological and pathological conditions has been previously reported [30–33]. In our recent study, we observed that in patients with cerebral vasospasm after aneurysmal subarachnoid hemorrhage BRS was “stunned” almost 3 weeks after aneurysm rupture [33], indicating short-term changes in BRS values. Changes in BRS over longer periods of time have also been described in prior research which suggested that in physiological conditions baroreflex function may exhibit a circadian rhythm [34] and may change during the reproductive cycle and during pregnancy [30, 32].

We found an association between temporal patterns of BRS and prognosis in the days following TBI. In patients with good outcome, there was a significant but weak increase in BRS, with temporal changes in BRS showing either a “U-shaped” curve or a gradual increase over time. BRS at 1.5 days after TBI was a significant predictor of mortality with better accuracy than PRx. Also, in patients who died BRS gradually decreased or remained low during a rise in ICP. Additional results concerning daily time trends of ICP and BRS in deceased patients showed uncoupling between rising ICP and BRS, as BRS remained low during a rise in ICP. The results of this study indicate that in TBI patients, BRS may likewise be a time-dependent parameter, and the dynamics of changes in BRS in the early days after TBI may predict mortality.

### BRS as a time-dependent parameter

In this study, we observed very short-term variations in BRS in the days following TBI that carry a potential prognostic significance. In patients with good outcome, BRS progressively increased after it has initially reached a lower breakpoint (“U-shaped” curve). This characteristic pattern was observed both in individual daily time trends of BRS in each patient and during the investigation of average breakpoints of BRS in the full group. In patients with poor outcome, dynamic changes in BRS were not found. In patients who died, we identified an “unfavorable” pattern in BRS time trends: either BRS remained relatively low (below 5 ms/mm Hg) and did not significantly change, or it decreased progressively in the first few days. Thus, daily observation of individual dynamics of changes in BRS may provide additional information about patients’ prognosis. Moreover, BRS assessment can be readily available for intensive care units, as it is based on continuous monitoring of ABP and HR which is frequently applied in routine care.

### BRS dynamics as a predictor of mortality

Our observation of the association between low BRS and mortality is consistent with findings from previous studies. Sykora et al. [18] found mean BRS to be low in non-survivors after TBI. Another study by Papaioannou et al, which presented the longitudinal alterations in ANS metrics over time in 20 brain injury patients, showed that BRS progressively and significantly increased in survivors. In the same study, no significant alterations in BRS over time were observed in brain death patients [17].

Based on the results of our study, the occurrence of an “unfavorable” pattern in BRS time trends should be considered as a potential warning sign for increased risk of mortality. We found that the mean value of BRS <= 1.8 ms/mm Hg estimated at 1.5 days after TBI is a better early predictor of mortality in terms of accuracy and specificity than PRx >= 0.17 (an autoregulation index) assessed during the same period and GCS <= 8 evaluated at admission. Compared to GCS, both BRS and PRx achieved better specificity, which means that they could allow for a more precise selection of patients with an increased risk of death. Concerning late prediction of mortality, the occurrence of an “unfavorable” pattern in BRS time trends had comparable sensitivity and specificity as mean PRx > 0.3 when estimated during the week that followed TBI.

In clinical practice, it may be useful to determine a critical moment of worsening of cerebral hemodynamics (e.g., assessed using PRx) and ANS (e.g., assessed using BRS). Pooled data analysis of the lower breakpoint of BRS during the week that followed TBI revealed that BRS reached a minimum about 2 days after TBI, with mean BRS at 1.5 days after brain injury significantly associated with mortality. PRx was found to be at its peak (indicating the worst cerebrovascular pressure reactivity) at 2.5 days after TBI, with mean PRx at day 1 significantly associated with mortality. Thus, an increase in BRS and a decrease in PRx 2 days after TBI might be considered as an indicator of good prognosis. While PRx has been more extensively studied in TBI compared to BRS, these two parameters should not be considered interchangeable but rather treated as complementary to each other as they reflect time-trends of cerebrovascular and autonomic cardiovascular parameters, respectively [18].

### Interaction of ICP with ANS activity

An important issue which needs to be addressed in the analysis of ANS in brain injury patients is rising ICP. It has been shown that a rise in ICP is related to a significant increase in BRS up to the upper breakpoint of the ICP amplitude–pressure characteristic, where mean ICP is at extreme levels, after which ICP continues to rise while BRS decreases [9]. Positive correlation between ICP and the high frequency component of HRV has been previously demonstrated in a study by Sykora et al. [18].

In our study, we found that BRS was significantly correlated with ICP when analyzed in the consequent 12-h windows. Based on the examined time trends we observed that changes in BRS parallel changes in ICP in patients with good outcome, in line with previous studies [8, 9, 11, 12]. Experiments performed with microneurography by Schmidt et al. [11] demonstrated that ICP is a determinant of efferent sympathetic outflow and that sympathetic activity increases with the rise in ICP. It was hypothesized that this served cerebral perfusion pressure at the cost of high systemic blood pressure. The study by Guild et al. [12] found that an increase in ICP is related to an increase in sympathetic drive and a rise in mean ABP, resulting in a relatively constant cerebral perfusion pressure. BRS, even though it mainly reflects parasympathetic activity, yields more information concerning the functioning of ANS. Preserved BRS requires a preserved afferent signal from the carotid bulb and aortic arch, as well as maintained central signal integration, yielding changes in HR and changes in peripheral resistance. The current preliminary study revealed that cerebrovascular reactivity and autonomic response are interlinked, with a bidirectional impact between cerebrovascular reactivity and circulatory autonomics [35].

The increase in ABP in patients with both good and poor outcomes may reflect the Cushing response to ICP rising, as most of the patients (90%) were in severe condition. Reduced HR in the poor outcome group, where 2/3 of patients had died, may follow a breakdown of the sympathetic part of ANS, as reported in a previous study by Sykora et al. where increased parasympathetic activity (increased high-frequency power of HRV) and decreased low-frequency-to-high-frequency ratio were observed in non-survivors [18].

### Relationship between baroreflex and cerebrovascular reactivity

The relationship between baroreflex and cerebrovascular reactivity is still unclear. None of the current cerebral hemodynamic models presented in the literature [36–38] can mimic the relationship between heart rate and ICP or PRx. None of them also attempt to simulate BRS. In a recent study, Froese et al. [35] used impulse response function plots to demonstrate that changes in cerebrovascular reactivity resulting from changes in BRS and HRV parameters were larger than the BRS and HRV response produced by changes in cerebrovascular reactivity; however, these observations were only statistically significant for patients with a Marshall CT score of 4 or higher. Based on Granger causality testing, the authors found that BRS has a meaningful directional impact on PRx only in 10% of patients, which reflects the fact that the interrelationship between cerebrovascular reactivity and ANS is heterogeneous and varies from patient to patient. In earlier studies, it has been hypothesized that cerebrovascular autoregulation impairment may be related to autonomic failure complicating TBI [39]. In our study, we observed that patients with better cerebral autoregulation and better BRS had better outcome. On the contrary, persistent impairment of cerebral autoregulation along with worsening BRS in the acute phase of TBI was a poor prognostic factor. This suggests that cerebral autoregulation and BRS are complementary to each other and provide a more complex picture of the impairment of cerebral blood flow regulation. BRS assessment offers the advantage of easier monitoring whereas PRx requires invasive ICP monitoring which is not available or required in patients with less severe TBI.

### Limitations

This study was conducted as a retrospective analysis of a relatively small set of patient data. However, the management protocol was the same for all patients which potentially limited variability. Propofol does have an effect on the ANS, but the significance of this influence is still under investigation. In the study of Mendez et al., the authors found that in normotensive subjects BRS and the gain of the transfer function between systolic blood pressure and RR interval in the low frequency band were significantly reduced after propofol injection and in post-intubation periods [40]. In another study of Porta et al., the authors found that the proposed model-based closed-loop approach detected a decrease in BRS after the injection of propofol anaesthesia [41]. Propofol has also been reported to reduce sympathetic autonomic outflow and decrease ABP as a result of its vasodilatory effect [42]. In our study, we had a group of patients who received propofol in a dose of 1.2 to 2.3 mg/kg/h and we assessed BRS in those patients under sedation. Propofol most probably buffers baroreflex activity and may influence the absolute value of BRS. As a consequence, this might have increased the magnitude of the effect that we observed concerning BRS being reduced with no dynamics towards an increase of this parameter in the first days following TBI in patients with poor prognosis. This limitation, however, is common to studies done in intensive care. Furthermore, it is possible that our approach of estimating BRS time trends during long monitoring permitted, owing to the accumulation of data, to reach better precision in the assessment of BRS dynamics, as compared to previous studies that relied on selected short time windows free from interventions and confounding medications. Our hypothesis is that the information from longer recordings yields information on BRS dynamics that outweighs the transient changes and potential confounding factors that are associated with critical care environment. It also permits to assess BRS dynamics in “real-life” conditions. Another confounding factor is mechanical ventilation. A previous study has shown that mechanical ventilation can attenuate respiratory arrhythmia and alter BRS [43]. In our study, due to severe TBI in 90% of patients, all of the patients were mechanically ventilated. Lung-protective ventilation was used, aimed at using low tidal volumes with optimum positive end-expiratory pressures set at 7 cm H_2_O with 4–6 mL/kg tidal volume ventilation. However, this could have been another potential confounding factor. Unfortunately, end-tidal CO_2_ (EtCO_2_) data was not continuously monitored. Although PaCO_2_ in arterial blood was within the normal range, i.e., 35–45 mm Hg in all patients, we cannot rule out that EtCO_2_ differed between patients and could have influenced the results of PRx assessment. However, the impact of EtCO_2_ on PRx is larger than its limited influence on BRS which was the main endpoint of this study.

## Conclusions

We found an association between temporal patterns of BRS and prognosis in the days following TBI. The changes in BRS over a long time period have a prognostic value in terms of outcome and mortality. In the short-term, when measured daily, BRS may reflect the changes in ANS related to the severity of TBI. Further research in a large multi-center study is needed to confirm these preliminary findings on the association between time trends of BRS and prognosis in TBI patients.

## Supplementary information

ESM 1. (DOCX 16 kb)
