# Supplementary material for: Association between temporal patterns of baroreflex sensitivity after traumatic brain injury and prognosis: a preliminary study
Source: Neurol Sci. 2023 Jan 7;44(5):1653–63. doi: 10.1007/s10072-022-06579-7 (PMC10102132; doi:10.1007/s10072-022-06579-7)
Supplement: Supplementary file 1 — (DOCX 16 kb) [file 10072_2022_6579_MOESM1_ESM.docx]

**Supplementary Table 1**

Median values and interquartile ranges of baroreflex sensitivity (BRS), intracranial pressure (ICP) and pressure reactivity index (PRx) in the full group of traumatic brain injury patients averaged over consecutive non-overlapping 12-hour windows in the first seven days after injury

|  | Total group | | |
| --- | --- | --- | --- |
| Day | BRS [ms/mm Hg] | ICP [mm Hg] | PRx [a.u.] |
| 0.5 | 2.29 ± 3.49 | 1.95 ± 1.19 | 0.31 ± 0.38 |
| 1 | 3.07 ± 4.16 | 1.87 ± 1.29 | 0.18 ± 0.27 |
| 1.5 | 4.15 ± 3.98 | 3.51 ± 10.98 | 0.12 ± 0.52 |
| 2 | 3.70 ± 5.17 | 4.22 ± 10.97 | 0.22 ± 0.53 |
| 2.5 | 4.69 ± 3.00 | 9.59 ± 11.80 | 0.13 ± 0.38 |
| 3 | 5.09 ± 4.66 | 8.31 ± 9.75 | 0.08 ± 0.31 |
| 3.5 | 5.56 ± 3.91 | 7.69 ± 9.98 | 0.08 ± 0.33 |
| 4 | 5.36 ± 4.23 | 8.94 ± 10.65 | 0.08 ± 0.35 |
| 4.5 | 5.53 ± 5.31 | 9.64 ± 8.64 | -0.02 ± 0.39 |
| 5 | 6.23 ± 5.52 | 8.91 ± 11.30 | 0.08 ± 0.45 |
| 5.5 | 4.22 ± 7.39 | 8.13 ± 9.98 | 0.12 ± 0.20 |
| 6 | 6.36 ± 5.11 | 8.95 ± 8.85 | 0.15 ± 0.28 |
| 6.5 | 6.38 ± 6.73 | 10.49 ± 8.03 | 0.27 ± 0.22 |
| 7 | 7.30 ± 6.65 | 9.81 ± 6.74 | 0.25 ± 0.25 |
|  | Poor outcome | | |
| Day | BRS [ms/mm Hg] | ICP [mm Hg] | PRx [a.u.] |
| 0.5 | 2.09 ± 0.83 | 1.35 ± 1.37 | 0.33 ± 0.47 |
| 1 | 2.32 ± 6.68 | 1.57 ± 1.32 | 0.40 ± 0.61 |
| 1.5 | 3.69 ± 4.19 | 4.74 ± 10.54 | 0.16 ± 0.17 |
| 2 | 3.53 ± 3.89 | 7.70 ± 12.18 | 0.32 ± 0.47 |
| 2.5 | 5.12± 3.79 | 12.82 ± 11.28 | 0.34 ± 0.52 |
| 3 | 5.09 ± 4.66 | 11.19 ± 10.81 | 0.25 ± 0.53 |
| 3.5 | 6.39 ± 3.02 | 10.63 ± 11.39 | 0.22 ± 0.27 |
| 4 | 5.03± 3.79 | 13.42 ± 10.98 | 0.19 ± 0.30 |
| 4.5 | 5.06 ± 4.53 | 9.04 ± 7.18 | 0.08 ± 0.60 |
| 5 | 5.81 ± 3.97 | 6.91 ± 6.99 | 0.22 ± 0.49 |
| 5.5 | 3.81 ± 6.15 | 6.52 ± 8.74 | 0.07 ± 0.20 |
| 6 | 3.34 ± 4.26 | 6.07 ± 6.95 | 0.27 ± 0.22 |
| 6.5 | 6.33 ± 6.00 | 9.82 ± 7.19 | 0.30 ± 0.09 |
| 7 | 5.53 ± 6.00 | 8.52 ± 10.10 | 0.30 ± 0.11 |
|  | Good outcome | | |
| Day | BRS [ms/mm Hg] | ICP [mm Hg] | PRx [a.u.] |
| 0.5 | 2.87 ± 3.86 | 2.08 ± 0.71 | 0.24 ± 0.35 |
| 1 | 3.96 ± 3.37 | 2.05 ± 0.96 | 0.15 ± 0.17 |
| 1.5 | 4.47 ± 3.80 | 3.13 ± 9.24 | 0.09 ± 0.60 |
| 2 | 4.04 ± 6.24 | 3.51 ± 10.59 | -0.01 ± 0.57 |
| 2.5 | 3.73 ± 2.68 | 6.19 ± 9.60 | 0.02 ± 0.26 |
| 3 | 5.13 ± 4.90 | 4.81 ± 7.70 | 0.04 ± 0.22 |
| 3.5 | 4.66 ± 6.59 | 5.35 ± 8.16 | 0.03 ± 0.26 |
| 4 | 5.81 ± 5.53 | 6.51 ± 7.03 | -0.01 ± 0.31 |
| 4.5 | 6.32 ± 8.90 | 9.68 ± 9.12 | -0.02 ± 0.35 |
| 5 | 8.25 ± 9.01 | 9.47 ± 11.81 | 0.07 ± 0.41 |
| 5.5 | 5.03 ± 7.71 | 8.13 ± 12.46 | 0.13 ± 0.20 |
| 6 | 6.71 ± 5.41 | 11.09 ± 11.82 | 0.09 ± 0.36 |
| 6.5 | 6.99 ± 7.92 | 14.27 ± 10.10 | 0.17 ± 0.35 |
| 7 | 9.29 ± 6.27 | 10.88 ± 3.71 | 0.19 ± 0.38 |

**Supplementary Table 2**

Cut-off values of baroreflex sensitivity (BRS) and pressure reactivity index (PRx) to predict mortality in traumatic brain injury patients based on receiver operating characteristic curve analysis. Values of BRS and PRx were averaged in 12-hour non-overlapping windows.

|  | BRS | | | PRx | | |
| --- | --- | --- | --- | --- | --- | --- |
| Day | Cut-off value | AUC | p-value | Cut-off value | AUC | p-value |
| 0.5 | 1.26 | 0.61 | 0.591 | 0.65 | 0.69 | 0.313 |
| 1 | 2.50 | 0.67 | 0.425 | 0.23 | 0.92 | **<0.001** |
| 1.5 | 1.82 | 0.83 | **<0.001** | 0.17 | 0.77 | **0.026** |
| 2 | 1.63 | 0.65 | 0.312 | 0.30 | 0.81 | **0.001** |
| 2.5 | 1.37 | 0.38 | 0.345 | 0.35 | 0.81 | **0.001** |
| 3 | 1.47 | 0.42 | 0.529 | 0.21 | 0.75 | **0.025** |
| 3.5 | 2.09 | 0.45 | 0.686 | 0.11 | 0.68 | 0.128 |
| 4 | 1.89 | 0.56 | 0.679 | 0.09 | 0.61 | 0.428 |
| 4.5 | 1.89 | 0.56 | 0.656 | 0.19 | 0.54 | 0.802 |
| 5 | 1.65 | 0.71 | 0.067 | 0.37 | 0.63 | 0.391 |
| 5.5 | 2.48 | 0.69 | 0.171 | -0.05 | 0.27 | 0.106 |
| 6 | 2.87 | 0.73 | 0.256 | 0.22 | 0.43 | 0.717 |
| 6.5 | 1.84 | 0.70 | 0.235 | 0.30 | 0.78 | 0.063 |
| 7 | 5.60 | 0.75 | 0.170 | 0.21 | 0.50 | 0.998 |
